# Supplementary material for: A neural basis for brain leptin action on reducing type 1 diabetic hyperglycemia
Source: Nat Commun. 2021 May 11;12:2662. doi: 10.1038/s41467-021-22940-4 (PMC8113586; doi:10.1038/s41467-021-22940-4)
Supplement: Supplementary file 1 — Supplementary Information [file 41467_2021_22940_MOESM1_ESM.pdf]

Supplementary Information for

A Neural Basis for Brain Leptin Action on Reducing Type 1 Diabetic Hyperglycemia

by

Shengjie Fan, Yuanzhong Xu, Yungang Lu, Zhiying Jiang, Hongli Li, Jessie Morrill, Jing Cai, Qi Wu, Yong Xu, Mingshan Xue, Benjamin R. Arenkiel, Cheng Huang and Qingchun Tong

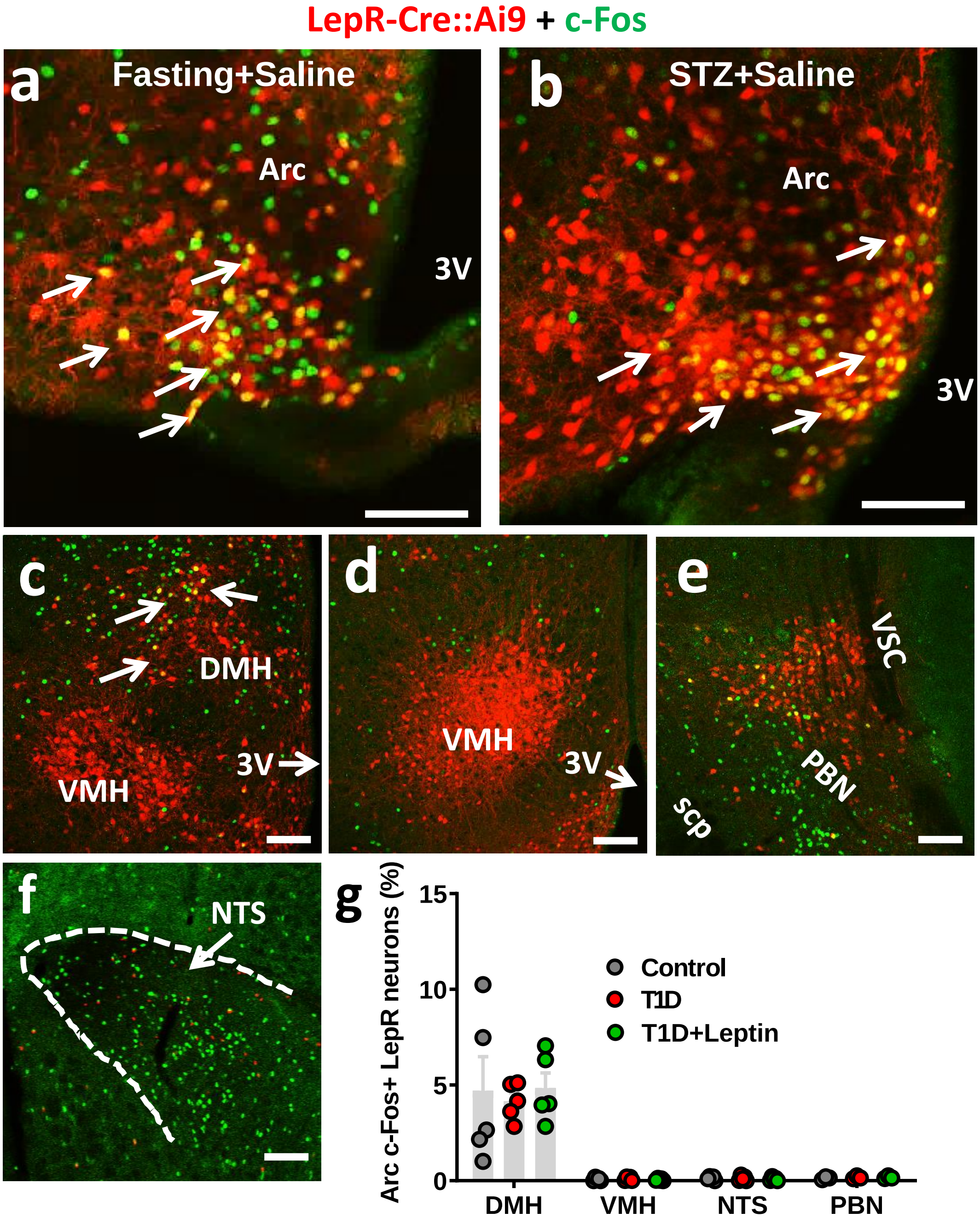

Supplementary Fig. 1. Selective activation of Arc LepR neurons in type 1 diabetes (T1D) within all LepR neurons. Related to Figure 1. (a-b) High magnification pictures showing colocalization of c-Fos (green) and LepR neurons (red) in the Arc in response to fasting (a) and ad libitum fed T1D (b). (c-f) In fed T1D mice, expression of c-Fos (green) in LepR neurons (red) in other brain sites, which are known to play a role in fasting-responses including DMH (c), VMH (d), PBN (e) and NTS (f). (g) comparison in percentage of LepR neurons that express c-Fos in these brain regions (n=4).

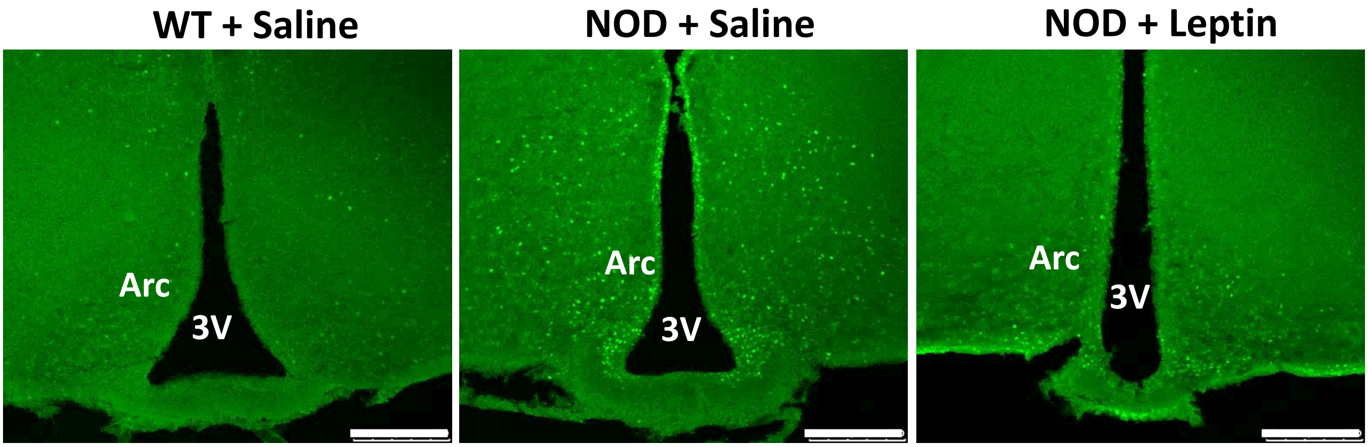

Supplementary Fig. 2. Activation of Arc neurons in non-obese diabetes (NOD) mice, related to Figure 1. Representative pictures showing c-Fos expression in the Arc of controls (left panels), NOD with i.c.v. saline treatment (middle panels) and NOD with i.c.v. leptin treatment (right panels).

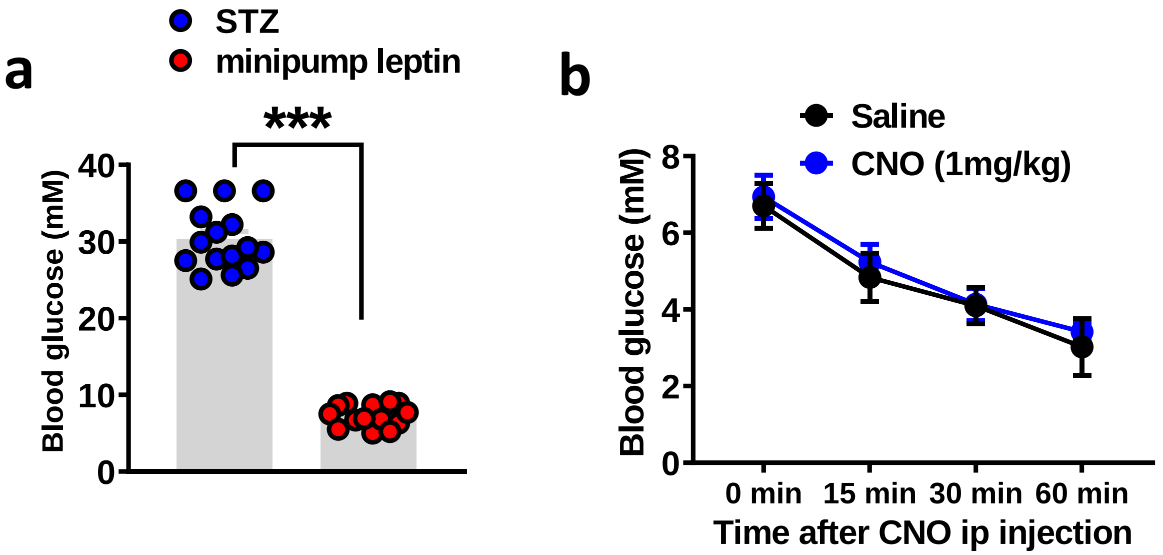

Supplementary Fig. 3. **CNO alone has no effect on glucose levels**. a) a group of C57 male mice (8-10 weeks old) were T1D hyperglycemia after STZ treatment, and glucose levels before (blue) and after i.c.v. leptin treatment (red) were shown (2-tailed unpaired Student's t tests, t=20.53, df=27, ***p<0.0001). b) The same group of mice treated with leptin were randomly divided in 2 groups, one treated with saline and the other with CNO (1mg/kg) and changes in glucose levels were shown (two-way ANOVA, n=5 for saline and 6 for CNO, F(2, 91)=10.4, p=0.9731, saline vs CNO at the 60 min time point).

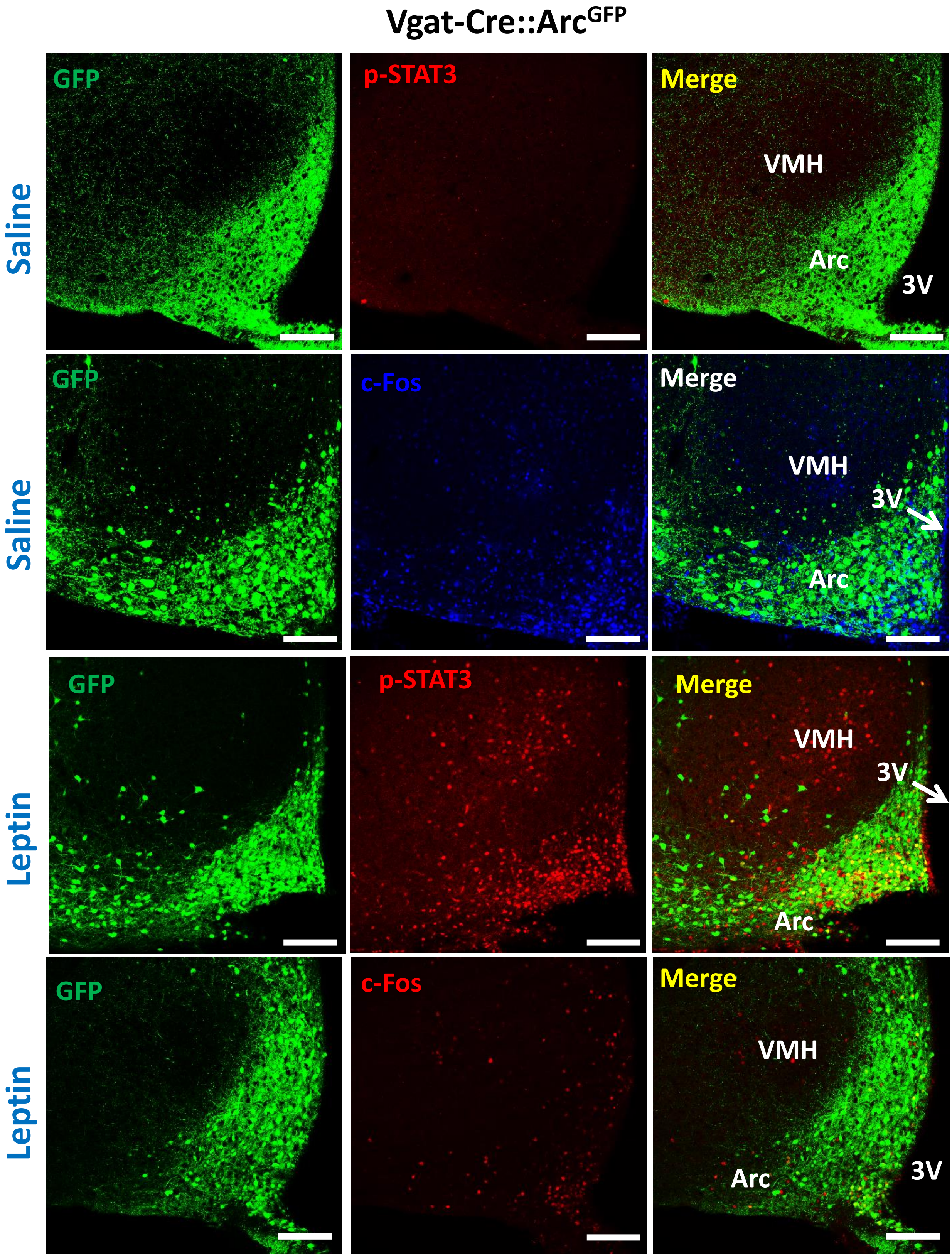

Supplementary Fig. 4. Leptin effectively reduced c-Fos in GABA+ neurons of Arc in type 1 diabetes (T1D) mice. Vgat-Cre mice were delivered AAV-Flex-GFP viral vectors to the Arc, treated with STZ to induce T1D and then received i.c.v. treatment of either saline and leptin at indicated. Expression of GFP (left panels), p-STAT3 or c-Fos as indicated (middle panels) and respective merged pictures (right panels). Scale bar = 100μM, Arc: arcuate nucleus; VMH: ventromedial hypothalamus.

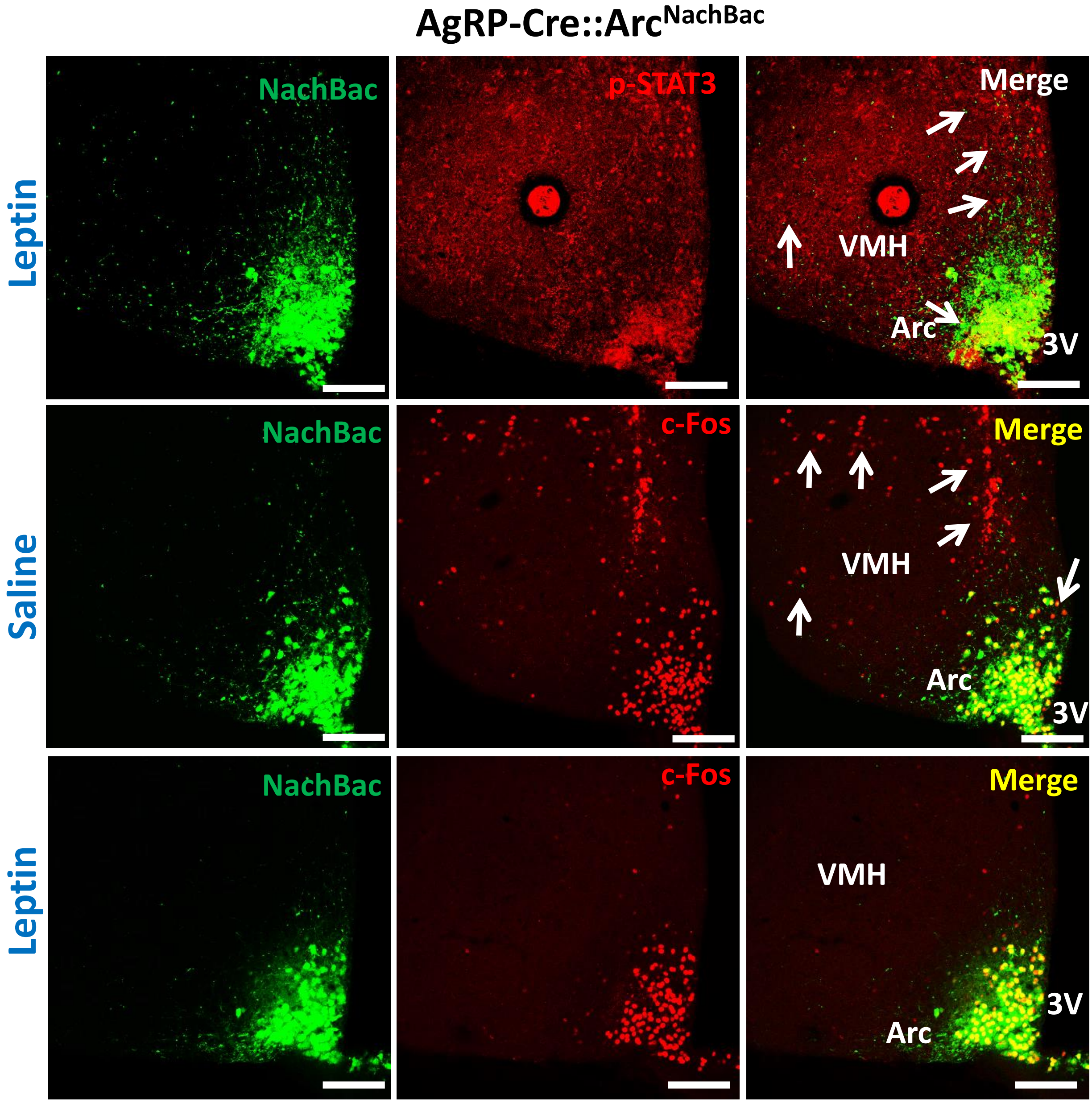

Supplementary Fig. 5. Leptin failed to reduce the activity of AgRP neurons with NachBac expression in T1D mice. AgRP-Cre mice were delivered AAV-Flex-NachBac-GFP viral vectors to the Arc, treated with STZ to induce T1D and then received i.c.v. treatment of either saline and leptin at indicated. Expression of GFP (left panels), p-STAT3 or c-Fos as indicated (middle panels) and respective merged pictures (right panels). Scale bar = 100µM, Arc: arcuate nucleus; VMH: ventromedial hypothalamus.

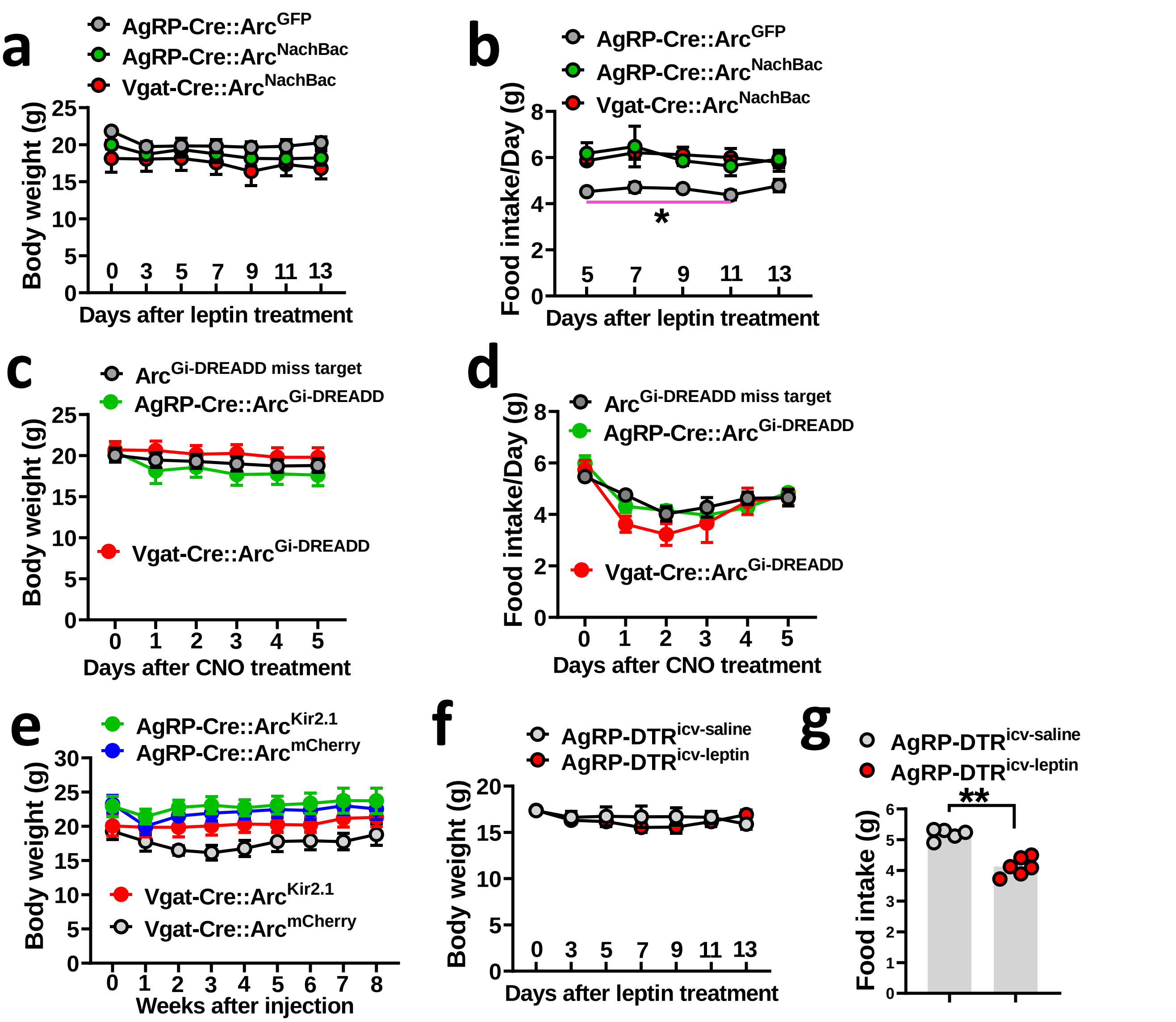

Supplementary Fig. 6. Changes in body weight and food intake in the studied mouse groups with changes in neuron activity. a-b) Changes in body weight (a, two-way ANOVA, n=7 for AgRP-GFP, 5 for AgRP-NachBac and 4 for Vgat-NachBac, $F(2,91)=10.4$, $p=0.3348$, AgRP-GFP vs AgRP-NachBac; $p=0.0701$, AgRP-GFP vs Vgat-NachBac; and $p=0.6717$, AgRP-NachBac vs Vgat-NachBac at day 13) and food intake (b, two-way ANOVA, n=7 for AgRP-GFP, 5 for AgRP-NachBac and 4 for Vgat-NachBac, $F(2,60)=26.28$, $p=0.0592$, AgRP-GFP vs AgRP-NachBac; $p=0.144$, AgRP-GFP vs Vgat-NachBac; and $p=0.9624$, AgRP-NachBac vs Vgat-NachBac at day 13) in T1D mice with leptin treatment and Arc Vgat or AgRP neurons expression of NachBac; c-d) Changes in body weight (c, two-way ANOVA, n=5/each, $F(2,72)=4.212$, $p=0.7956$, missed vs Vgat-Gi-DREADD; and $p=0.7355$, missed vs AgRP-Gi-DREADD) and food take (d, two-way ANOVA, n=5/each, $F(2,72)=2.496$, $p=0.9986$, missed vs Vgat-Gi-DREADD; and $p=0.9095$, missed vs AgRP-Gi-DREADD) in T1D mice with Arc Vgat or AgRP neuron expression of Gi-DREADD; e) Changes in body weight in T1D mice with AgRP or Vgat neuron expression of Kir2.1 (two-way ANOVA, t=7 for Vgat-Kir2.1 and AgRP-Kir2.1; 6 for Vgat-mCherry and 5 for AgRP-mCherry, $F(3, 189)=28.31$, $p=0.5306$, Vgat-Kir2.1 vs Vgat-mCherry; and $p=0.9259$, AgRP-Kir2.1 vs AgRP-mCherry) at day 8; and f-g) Changes in body weight (f, two-way-ANOVA, t=5/each, $F(1, 56)=1.000$, $p=0.8892$, Saline vs DTX) and food intake (g, 2-tailed upaired Student's t tests, n=5/each, $t=6.964$, df=9; **$p<0.0001$) in AgRP neuron lesioned mice (AgRP-DTR) treated with saline or leptin.

a

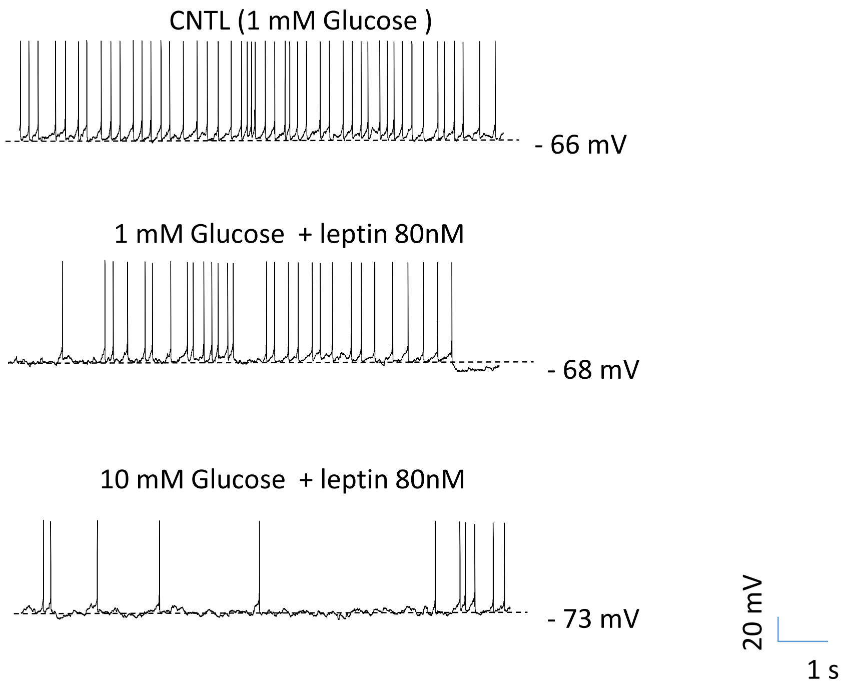

b

Data Summary

	Glucose	Leptin	Both
**Inhibited #**	**71 (80%)**	**73 (84%)**	**50 (67.5%)**
**Excited #**	**18 (20%)**	**14 (16%)**	**6 (8%)**
**Sum#**	**89**	**87**	**74**

Supplementary Fig 7. Leptin inhibition of Arc LepR neurons depends on glucose levels, related to Figure 6. (a) Representative traces from one recorded LepR neuron showing that the inhibitory action of leptin of firing rates is enhanced by increasing glucose levels from 1mM to 10 mM. (b) Overall summary of all LepR neurons in the Arc that were recorded.
