# Supplementary material for: A neural basis for brain leptin action on reducing type 1 diabetic hyperglycemia
Source: Nat Commun. 2021 May 11;12:2662. doi: 10.1038/s41467-021-22940-4 (PMC8113586; doi:10.1038/s41467-021-22940-4)
Supplement: Supplementary file 3 — Reporting Summary [file 41467_2021_22940_MOESM3_ESM.pdf]

natureresearch

Corresponding author(s): Qingchun Tong

Last updated by author(s): Apr 1, 2021

Reporting Summary

Nature Research wishes to improve the reproducibility of the work that we publish. This form provides structure for consistency and transparency in reporting. For further information on Nature Research policies, see Authors & Referees and the Editorial Policy Checklist .

Statistics

For all statistical analyses, confirm that the following items are present in the figure legend, table legend, main text, or Methods section.

n/a	Confirmed	
☐	☒	The exact sample size (*n*) for each experimental group/condition, given as a discrete number and unit of measurement
☐	☒	A statement on whether measurements were taken from distinct samples or whether the same sample was measured repeatedly
☐	☒	The statistical test(s) used AND whether they are one- or two-sided<br>*Only common tests should be described solely by name; describe more complex techniques in the Methods section.*
☐	☒	A description of all covariates tested
☐	☒	A description of any assumptions or corrections, such as tests of normality and adjustment for multiple comparisons
☐	☒	A full description of the statistical parameters including central tendency (e.g. means) or other basic estimates (e.g. regression coefficient) AND variation (e.g. standard deviation) or associated estimates of uncertainty (e.g. confidence intervals)
☐	☒	For null hypothesis testing, the test statistic (e.g. *F*, *t*, *r*) with confidence intervals, effect sizes, degrees of freedom and *P* value noted<br>*Give P values as exact values whenever suitable.*
☒	☐	For Bayesian analysis, information on the choice of priors and Markov chain Monte Carlo settings
☒	☐	For hierarchical and complex designs, identification of the appropriate level for tests and full reporting of outcomes
☒	☐	Estimates of effect sizes (e.g. Cohen's *d*, Pearson's *r*), indicating how they were calculated

Our web collection on statistics for biologists contains articles on many of the points above.

Software and code

Policy information about availability of computer code

Data collection	Microsoft Excel 2020, GraphPad Prism 8 and pCLAMP10
Data analysis	GraphPad Prism 8 and Adobe Photoshop CS6.

For manuscripts utilizing custom algorithms or software that are central to the research but not yet described in published literature, software must be made available to editors/reviewers. We strongly encourage code deposition in a community repository (e.g. GitHub). See the Nature Research guidelines for submitting code & software for further information.

Data

Policy information about availability of data

All manuscripts must include a data availability statement. This statement should provide the following information, where applicable:

- Accession codes, unique identifiers, or web links for publicly available datasets
- A list of figures that have associated raw data
- A description of any restrictions on data availability

All data generated in this study are available and reported. We will provide raw data on those figures that are not provided with individual data points. We will also provide all information on statistical analysis including effects size, degree of freedom and exact p values.

Field-specific reporting

Please select the one below that is the best fit for your research. If you are not sure, read the appropriate sections before making your selection.

☒ Life sciences ☐ Behavioural & social sciences ☐ Ecological, evolutionary & environmental sciences

Life sciences study design

All studies must disclose on these points even when the disclosure is negative.

Sample size	Sample size (n=3-9) was predetermined based on literature and our own experience in body weight studies. Given the dramatic difference in glucose levels observed in most of our studies, some of the study groups used n> or = 5. We used n=3/group for all c-Fos comparison studies, which is normally used in related literature and is sufficient to reveal difference. For electrophysiologic studies, we used neuron number n=13-21. These neurons were from 3-4 animals each group. These numbers are within the ranges of similar studies in the related literature. Details were described in Figure Legends.
Data exclusions	Most of data collected used for analysis. The exception is that, after posthoc analysis on AAV vector delivery, those with evidence of off-targeting, i.e. a significant number of vector expression outside the Arc were excluded for body weight analysis.
Replication	Due to long time required for each stereotaxic surgery, study group subjects of each group were performed during different days with differnet experiments. Results from these different experiments were the same and combined.
Randomization	All animals with same genotype were randomly divided into control or experimental groups.
Blinding	No blinding procedure was implemented. However, the researcher who measured body weight was not aware of mouse grouping information.

Reporting for specific materials, systems and methods

We require information from authors about some types of materials, experimental systems and methods used in many studies. Here, indicate whether each material, system or method listed is relevant to your study. If you are not sure if a list item applies to your research, read the appropriate section before selecting a response.

Materials & experimental systems

n/a	Involved in the study	
☐	☒	Antibodies
☒	☐	Eukaryotic cell lines
☒	☐	Palaeontology
☐	☒	Animals and other organisms
☒	☐	Human research participants
☒	☐	Clinical data

Methods

n/a	Involved in the study	
☒	☐	ChIP-seq
☒	☐	Flow cytometry
☒	☐	MRI-based neuroimaging

Antibodies

Antibodies used	1. Anti-c-Fos Antibody; Millipore; ABE457,Species Reactivity:H, R; LOT# 2552627<br>2. Phospho-Stat3 (Tyr705) (D3A7) XP Rabbit mAb;Cell signaling technology;#9145; Reactivity:H M R Mk; LOT # 18<br>3. Phospho-AMPKα (Thr172) Antibody; Cell signaling technology; #2531; Reactivity:H M R Mk; LOT # 15<br>4. AlexaFluor 488 conjugated donkey anti-rabbit IgGs; Jackson ImmunoResearch Laboratories; Cat#: 711-547-003; LOT # 110898
Validation	All antibodies have been used and verified in the literature, and also verified by our own results with expected changes in control animals. C-Fos antibody has been used in Alvisi RD et al., Life Sciences, 2016; The p-STAT3 antibody has been widely used by others with more than 1000 citations including Bromberg JF et al., Cell: 98: 295-303, 1999; p-AMPKa antibody has also been widely used with more than 600 citations including Hardie DG et la., J. Cell Sci.: 117: 479-87, 2014; and the AlexaFluo 488 secondary antibody has been used by others including Tai-Nagara I et al., Nature Communications, 2020.

Animals and other organisms

Policy information about studies involving animals; ARRIVE guidelines recommended for reporting animal research

Laboratory animals	All mice were housed with ad libitum access to water and food in a temperature controlled room (21°C-22°C) with a 12:12 hour light-dark cycle. Animal care and procedures were approved by the Animal Welfare Committee of The University of Texas Health Science Center at Houston. Vgat-Cre or AgRP-Cre mice from the Jax lab and were bred to Ai9 red reporter or (ROSA)26Sortm1.1 (CAG-cas9*,-EGFP) green reporter to get generate respective reporter mice for electrophysiological recording, assessing viral delivery quality or colocalization with c-Fos immunohistochemistry. AgRP-DTR mice were provided by Dr. Qi Wu and verified in this study. These animals are on mixed background and bred with Vgat-Cre mice. All mice were used from 7 weeks old to 20 weeks old of age, except for AgRP-DTR and the associated compound mice, which received DTX injections at days 3 and 5. Most of studies used males and some of the recording studies used females.
Wild animals	No wild animals were used in this study.

Field-collected samples	No field-collected samples were used in this study.
Ethics oversight	All animal studies have been approved by the Animal Welfare Committee of University of Texas Health Science Center at Houston or Shanghai University of Traditional Chinese Medicine.

Note that full information on the approval of the study protocol must also be provided in the manuscript.
